# Direct production of itaconic acid from liquefied corn starch by genetically engineered *Aspergillus terreus*

**DOI:** 10.1186/s12934-014-0108-1

**Published:** 2014-08-17

**Authors:** Xuenian Huang, Mei Chen, Xuefeng Lu, Yueming Li, Xia Li, Jian-Jun Li

**Affiliations:** Key Laboratory of Biofuels, Shandong Provincial Key Laboratory of Energy Genetics, Qingdao Institute of Bioenergy and Bioprocess Technology, Chinese Academy of Sciences, No. 189 Songling Road, Qingdao, 266101 China; Qingdao Kehai Biochemistry Co, Ltd, No. 198 Langyatai Road, Jiaonan, 266400 Qingdao China; University of Chinese Academy of Sciences, Beijing, 100049 China

**Keywords:** *Aspergillus terreus*, Glucoamylase, Genetic engineering, Itaconate titer, Liquefied corn starch

## Abstract

**Background:**

Itaconic acid is on the DOE (Department of Energy) top 12 list of biotechnologically produced building block chemicals and is produced commercially by *Aspergillus terreus*. However, the production cost of itaconic acid is too high to be economically competitive with the petrochemical-based products. Itaconic acid is generally produced from raw corn starch, including three steps: enzymatic hydrolysis of corn starch into a glucose-rich syrup by α-amylase and glucoamylase, fermentation, and recovery of itaconic acid. The whole process is very time-consuming and energy-intensive.

**Results:**

In order to reduce the production cost, saccharification and fermentation were integrated into one step through overexpressing the glucoamylase gene in *A. terreus* under the control of the native *PcitA* promoter. The transformant XH61-5 produced higher itaconate titer from liquefied starch than WT. To further increase the titer by enhancing the secretion capacity of overexpressed glucoamylase, a stronger signal peptide was selected based on the major secreted protein ATEG_02176 (an acid phosphatase precursor) by *A. terreus* under the itaconate production conditions. Under the control of the stronger signal peptide, the transformant XH86-8 showed higher itaconate production level than XH61-5 from liquefied starch. The itaconate titer was further enhanced through a two-step process involving the vegetative and production phase, and the transformant XH86-8 produced comparable itaconate titer from liquefied starch to current one (~80 g/L) from saccharified starch hydrolysates in industry. The effects of the new signal peptide and the two-step process on itaconate production were investigated and discussed.

**Conclusions:**

Itaconic acid could be efficiently produced from liquefied corn starch by overexpressing the glucoamylase gene in *A. terreus*, which will be helpful for constructing a highly efficient microbial cell factory for itaconate production and for further lowering the production cost of itaconic acid.

**Electronic supplementary material:**

The online version of this article (doi:10.1186/s12934-014-0108-1) contains supplementary material, which is available to authorized users.

## Background

Itaconic acid (methylene succinic acid) is produced commercially by microbial fermentation using the fungus *A. terreus* [1]. It can be used in industry as monomer or co-monomer in the manufacturing of polymers, and is on the DOE (Department of Energy) top 12 list of the biotechnologically produced building block chemicals [1,2]. Particularly, it can even replace acrylic and methacrylic acid which is produced by the petrochemical industry [3]. However, according to the study of DOE, the production cost of itaconic acid needs to be reduced at least to $0.5/kg to be economically competitive with the present petrochemical-based products, which is currently not the case [1].

The highest itaconic acid yield is obtained when glucose is used as the substrate, but glucose is too expensive to be used as a raw material for commercial production of itaconic acid. Therefore, other raw materials that are cheaper than glucose, such as starch, molasses, hydrolysates of corn syrup or wood, were also tested. Among them, corn starch is one of the best carbon sources, since it is pure, relatively inexpensive, and stable in a mass supply [4–6].

Raw corn starch could not be directly used for itaconic acid production by *A. terreus* due to gelatinization upon heat sterilization [7]. Itaconic acid production from raw corn starch mainly includes three steps: enzymatic (or chemical) hydrolysis of corn starch into a glucose-rich syrup, fermentation, and recovery of itaconic acid [6–8]. Enzymatic hydrolysis of corn starch involves two steps: liquefaction in which corn starch is processed by α-amylase at round 106°C and saccharification where liquefied starch was further hydrolyzed into glucose by glucoamylaseat 60°C, which is very time-consuming and energy-intensive. Citric acid production from corn starch by *A. niger* follows similar procedures. However, given that *A. niger* secretes glucoamylase up to > 10 g/l [9], the saccharification step can be greatly cut down and even omitted [10]. What’s more, it has been reported that citric acid can be directly produced from corn and potato starch by 2-deoxyglucose-resistant mutant strains of *A. niger*, which showed the increased glucoamylase activity [10,11]. It seems that glucoamylases play important roles in direct production of citric acid from corn starch by *A. niger*.

Though there are lots of studies on itaconic acid production from starch hydrolysates [6,7], there have been no reports on direct production of itaconic acid from raw or liquefied or soluble starch by *A. terreus*. Instead, Kirimura *et al.* generated a fusant strain between *A. terreus* and *A. usamii* (a glucoamylase producer) producing maximally 35.9 g/L itaconic acid from soluble starch at day 6 of cultivation, whose productivity was five times as high as that of the parental *A. terreus* strain [12]. However, the itaconic acid titer is pretty low and the fermentation time is too long, so it is not suitable for industrial production of itaconic acid. In addition, it has been observed that addition of glucoamylases is beneficial for microbial production of itaconic acid using liquefied starch as the starting material [13]. Considering that purchasing glucoamylases incurs additional cost, ideally, *A. terreus* is genetically engineered to directly produce itaconic acid from liquefied starch through integrating saccharification and fermentation into one step, which would be achieved by overexpressing glucoamylases in *A. terreus*, thus direct production of itaconic acid from liquefied starch can be realized. To achieve this goal, at least three biological parts are required: an efficient promoter for glucoamylase overexpression in *A. terreus*, an appropriate glucoamylase gene and an appropriate signal peptide for high-level secretion of overexpressed glucoamylase.

The constitutive promoters which are not dependent on the carbon or nitrogen source or the specific inducers, and the native ones which are more efficient in directing gene expression than the heterologous ones, are preferred for genetic engineering [14,15]. So far, the only investigated native promoter from *A. terreus* is the *gpd* promoter, which has been successfully applied in driving *vgb* (the *Vitreoscilla* hemoglobin gene) overexpression in *A. terreus* [16]. *PgpdA* from *A. nidulans* was by far the most frequently used promoter for genetic modification of *A. terreus* [17,18]. In order to prevent squelching or titration of specific transcription factors, it is preferable to utilize multiple distinct promoters, with one specific promoter for each gene to be expressed [19,20]. Since citrate synthase is closely related to the itaconate precursor citric acid and plays an important role in biosynthesis of itaconic acid [1], the citrate synthase promoter from *A. terreus* is expected to be a strong constitutive and native promoter under the itaconic acid production conditions.

It is known that *A. niger* is an excellent glucoamylase producer, and can secrete glucoamylase up to >10 g/l [9]. Glucoamylase from *A. niger* has been commercialized and often used for saccharification [21].

Since glucoamylases need to be secreted out of the cells for efficient saccharification of liquefied starch, choosing the appropriate signal peptides is essential for high-level secretion of overexpressed glucoamylases [22].

In the current study, direct production of itaconic acid from liquefied starch by *A. terreus* was achieved by overexpressing glucoamylase from *A. niger* under the control of the native citrate synthase promoter, and the itaconate titer was further improved through using the native signal peptide of glucoamylase and optimizing the fermentation conditions. Therefore, an efficient microbial cell factory for direct production of itaconic acid from liquefied starch was established.

## Results

### Cloning, functional characterization, and deletion analysis of the citrate synthase promoter from *A. terreus*

A 1878-bp upstream sequence from the start codon (ATG) of the *citA* gene (ATEG_07990, encoding citrate synthase) of *A. terreus* CICC 40205 was cloned and sequenced, showing 94.7% sequence identity with the annotated one for ATEG_07990 in the genome sequence of *A. terreus* NIH 2624. In the upstream of *citA* from *A. terreus* CICC 40205, additional twelve nucleotide bases aaaaataaaaag were inserted between −398 and −409 from ATG (A as +1). The 1878-bp upstream sequence of the *citA* gene has been deposited at EBI (European Bioinformatics Institute) with the accession numberHG530534.

The promoter function of the putative promoter region of the *citA* gene was evaluated using *sgfp* (synthetic green fluorescent protein, codon optimized) as a reporter [23]. The expression cassette pXH31 for *PcitA1* (1878 bp, the full length of the putative *citA* promoter) was constructed and transformed into *A. terreus* CICC 40205 (Table 1). The bright field and fluorescent images of the randomly picked *A. terreus* transformant XH31-1 were taken in different stages: conidia, young hyphae, and mature hyphae (Additional file 1: Figure S1). As a negative control, the parental strain *A. terreus* CICC 40205 did not show any visible GFP fluorescence at all in these three stages (data not shown). These results clearly demonstrated that *PcitA1* successfully drove *sgfp* expression in *A. terreus* CICC 40205.

**Table 1:** **Transformants carrying different plasmids in this study**

**Variants**	**Integrated plasmid**	**Characteristics (randomly integrated)**
XH31	pXH31	*A. terreus*CICC 40205, *PcitA1-sgfp-TtrpC*, HmB^r^
XH32	pXH32	*A. terreus*CICC 40205, *PcitA2-sgfp-TtrpC*, HmB^r^
XH33	pXH33	*A. terreus*CICC 40205, *PcitA3-sgfp-TtrpC*, HmB^r^
XH34	pXH34	*A. terreus*CICC 40205, *PcitA4-sgfp-TtrpC*, HmB^r^
XH59	pXH59	*A. terreus*CICC 40205, *PcitA4-glaA-TtrpC*, HmB^r^
XH61	pXH61	*A. terreus*CICC 40205, *PcitA3-glaA-TtrpC*, HmB^r^
XH86	pXH86	*A. terreus*CICC 40205, *PcitA3-SP*_*02176*_ *-glaA1-TtrpC*, HmB^r^

*sgfp*: the gene encoding synthetic green fluorescent protein. *TtrpC*: *A. nidulans trpC* terminator. Hm B: hygromycin B resistance. *glaA*: the gene encoding glucoamylase from *A. niger. SP*_*02176*_: the gene encoding the signal peptide o facid phosphatase ATEG_02176.

In order to obtain the efficient and compact promoters which are preferable for genetic engineering and gene overexpression, three truncated ones *PcitA2* (−1121 bp from the start codon (ATG) of *citA*, A as +1), *PcitA3* (−610 bp from the start codon (ATG) of *citA*), and *PcitA4* (−262 bp from the start codon (ATG) of *citA*) were designed, among which *PcitA4* contains the three conserved stretches found in the putative promoter regions of *citA* gene from seven Aspergilli [19]. The corresponding expression cassettes pXH32, pXH33, and pXH34 were constructed and transformed into *A. terreus* CICC 40205 respectively (Additional file 2: Figure S2). Interestingly, the truncated promoters *PcitA2*, *PcitA3*, and *PcitA4* were also able to overexpress *sgfp* in *A. terreus* CICC 40205 in all three stages (Additional file 3: Figure S3). The fluorescent intensity of two randomly chosen transformants for each promoter were determined and compared according to a published procedure (Additional file 4: Figure S4) [16], and the results clearly demonstrated that four *citA* promoters with different lengths showed similar promoter activity. Therefore, two shorter ones *PcitA3* and *PcitA4* were applied for overexpression of glucoamylase in *A. terreus* in the following experiments.

### Cloning of the glucoamylase gene and overexpression in *A. terreus* under the control of *PcitA*

**Figure 1:**
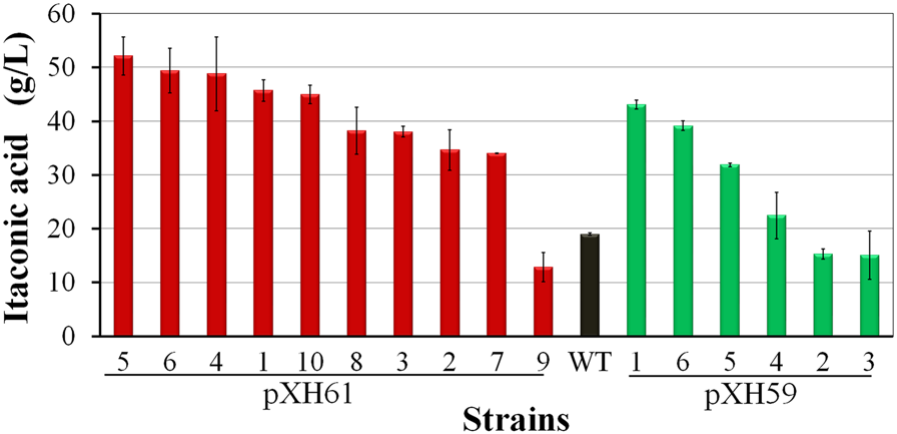
**Itaconic acid production from liquefied corn starch by the transformants of pXH61 and pXH59.** The transformants were screened using liquefied corn starch (corresponding to 140 g/L glucose) as the carbon source on a rotary shaker at 37°C for 72 h. Three independents experiments were performed for each strain. The itaconate titers were determined by HPLC.

The glucoamylase gene *glaA* (An03g06550) with its native signal peptide was cloned into pXH43 and pXH44 modified from pXH33 and pXH34 respectively (Additional file 5: Figure S5), which were transformed into *A. terreus* CICC 40205 separately. Ten transformants were tested for itaconic acid production. When saccharified corn starch hydrolysate was used as the carbon resource, all transformants showed similar itaconic acid production level to the parental strain (Additional file 6: Figure S6), indicating that introduction of the glucoamylase gene from *A. niger* into *A. terreus* CICC 40205 didn’t have adverse effect on itaconate production. By contrast, when they were further screened utilizing liquefied corn starch as the starting material, 13 out of 16 transformants produced higher itaconic acid titers than WT (Figure 1). What’s more, six transformants exhibited greatly improved itaconate production level than WT (Figure 1). For example, in comparison with WT (19.0 g/L), the transformant XH61-5 produced 52.1 g/L itaconic acid from liquefied corn starch (Table 1) (Figure 1).

These results demonstrated that: 1) the glucoamylase gene *gla*A from *A. niger* could be successfully overexpressed in *A. terreus* under the control of the native promoters *PcitA3* and *PcitA4*; 2) overexpressed glucoamylase could be effectively secreted out of cells by its native signal peptide; 3) overexpressed glucoamylase was active in saccharification of liquefied corn starch; 4) statistically (T-test, ρ = 0.038), *PcitA3* is better than *PcitA4* (Figure 1), which would be used in the following experiments. Thus, direct production of itaconic acid from liquefied corn starch through overexpression of glucoamylase in *A. terreus* is feasible.

### Selection and application of the stronger signal peptide

In order to enhance the secretion capacity of overexpressed glucoamylase, a strong signal peptide was chosen based on 2-DE (two-dimensional gel electrophoresis) of the secreted proteins by *A. terreus* CICC 40205 under the itaconate production conditions. The major proteins indicated by box (Additional file 7: Figure S7) with different isoelectric points, were identified to be ATEG_02176 (an acid phosphatase precursor) through HPLC-ESI-MS/MS, whose signal peptide was predicted to be MFSKQSLVSLLGGLSLALA using SignalP 3.0. Spots 1 and 2 were confirmed to be the fragments of ATEG_02176, and spot 3 could not be fully identified due to low score (Additional file 7: Figure S7).

**Figure 2:**
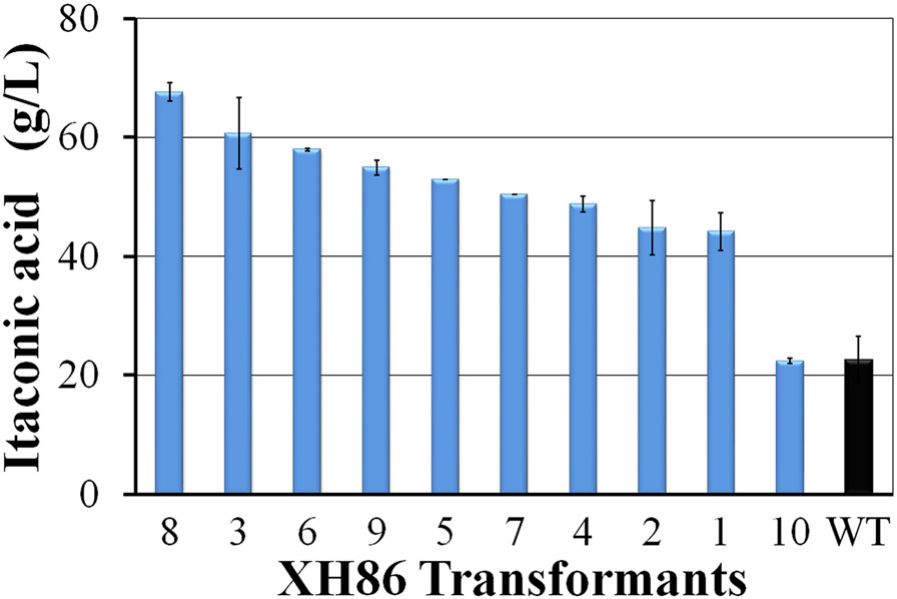
**Itaconic acid production from liquefied corn starch by the transformants of pXH86.** The transformants were screened using liquefied corn starch (corresponding to 140 g/L glucose) as the carbon source on a rotary shaker at 37°C for 72 h. Three independents experiments were performed for each strain. The itaconate titers were determined by HPLC.

The glucoamylase’s native signal peptide MSFRSLLALSGLVCTGLA was subsequently replaced by that of ATEG_02176. The new expression cassette pXH86 with the stronger signal peptide was constructed and transformed into *A. terreus* CICC 40205. Likewise, introducing the glucoamylase gene with the stronger signal peptide into *A. terreus* didn’t greatly impact itaconate production while saccharified corn starch hydrolysate was utilized as the carbon source (Additional file 8: Figure S8). When they were further tested using liquefied corn starch as the starting material, all transformants produced significantly higher itaconic acid titers than WT (Figure 2). The transformant XH86-8 was found to the best itaconate producer, whose itaconate titer from liquefied corn starch reached 67.6 g/L (Table 1) (Figure 2). These results showed that overexpressed glucoamylase could be efficiently secreted into the culture under the control of the signal peptide of ATEG_02176 and effectively hydrolyze liquefied corn starch into glucose. Statistically (T-test, ρ = 0.039), the signal peptide of ATEG_02176 is stronger than the native one of glucoamylase and beneficial for itaconate production (Figure 1 for XH61, Figure 2).

### Optimization of the fermentation conditions and comparison of the performance of WT and the transformants XH61-5 and XH86-8

In all screening experiments, no matter when saccharified corn starch hydrolysate or liquefied corn starch was utilized as the carbon source, the transformants were cultivated by direct inoculation of spores in itaconate production medium (IPM). To further improve the itaconic acid titer, a two-stage process including the vegetative (growing a pre-culture on saccharified corn starch hydrolysates) and fermentation (liquefied corn starch used as the substrate for further fermentation) phase was applied. The parental strain, and the transformants XH61-5 and XH86-8 were directly compared using liquefied corn starch as the carbon source in order to investigate the impact of the two-stage process as well as replacement of the signal peptide of gluoamylase on the performance of three strains, including growth, changes of residual glucose and oligosaccharides, itaconate titer, and glucoamylase production and activity.

According to the growth curves (Additional file 9: Figure S9), no matter whether the one-step process or the two-step one was used, the transformants XH61-5 and XH86-8 grew normally compared with WT growing from both liquefied corn starch and saccharified corn starch hydrolysates, demonstrating that introduction of the *glaA* gene didn’t have serious adverse effects on the growth of *A. terreus*. All three strains grew faster before 36 hr in the two-step process than in the one-step process respectively, and reached the stationary phase after 48 hr. The growth rates were determined based on these (Additional file 10: Table S1).

Based on the changes of glucose (Additional file 11: Figure S10), XH61-5 and XH86-8 showed similar trend in both processes: glucose gradually increased to the highest concentration, then gradually decreased to the lowest, but both reached the highest glucose concentration earlier in the two-stage process than in the one-stage process. WT exhibited same trend as XH61-5 and XH86-8 in the two-stage processes, but different behaviour in the one-stage process: the glucose concentration gradually decreased during the initial period, then increased all the time (Additional file 11: Figure S10A,*inset*). It appears that WT experienced the stages of carbon starvation and adaptive response to carbon starvation, which might induce complex alterations in the transcriptome, including the expression of genes encoding important elements of primary and secondary metabolism and programmed cell death processes, and complex well-regulated and energy-consuming physiological and morphological changes [24–26]. As a consequence, itaconate production may be adversely affected (Figure 2). The highest glucose concentration was obtained for XH86-8 in both processes. The specific glucose formation rates were also determined (Additional file 10: Table S1). According to the changes of residual oligosaccharides (expressed by residual glucose equivalent) (Additional file 12: Figure S11), WT, XH61-5 and XH86-8 displayed same trend in both processes, and the consumption rates for oligosaccharides were XH86-8 > XH61-5 > WT and faster in the two-step process than in the one-step process. These results clearly demonstrated that overexpression of glucoamylase, the new signal peptide and the two-stage process are beneficial for hydrolysis of oligosaccharides.

**Figure 3:**
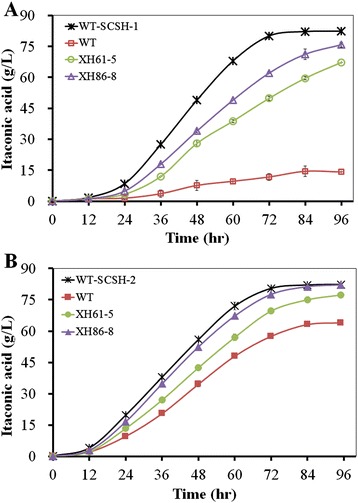
**Comparison of the effects of signal peptides on itaconate production from liquefied corn starch.** WT and the transformants XH61-5 and XH86-8 were compared in the one-step **(A)** and two-step **(B)** processes using liquefied corn starch (equivalent to 140 g/L glucose) as the carbon source. Cultures were sampled every 12 h. The itaconate titers were determined by HPLC. Itaconic acid production from saccharified corn starch hydrolysate (SCSH) by WT was used as the reference.

In the case of itaconic acid titers using liquefied corn starch as the carbon source, no matter whether the one-step process or the two-step one was utilized (Figure 3), the transformants XH61-5 and XH86-8 produced higher itaconate titers than WT, and XH86-8 exhibited higher itaconic acid production level than XH61-5, indicating that overexpression of glucoamylase and replacement of the signal peptide contributed greatly to improvement of itaconate titers. XH86-8 showed higher itaconate productivity in the two-step process than in the one-step process, demonstrating that the two-step process is helpful for itaconic acid production. Importantly, the itaconate titer of XH86-8 from liquefied corn starch in the two-step process is comparable to that of WT from saccharified corn starch hydrolysates (Figure 3B). The specific itaconate production rates were calculated according to these results (Additional file 10: Table S1).

As shown by SDS-PAGE (Additional file 13: Figure S12), overexpressed glucoamylase was clearly observed in the culture filtrate of XH86-8 in both processes, especially in the two–step process, but was not seen for WT in both processes and only a small amount of glucoamylase was observed for XH61-5 in both processes (data not shown). These results demonstrated that the new signal peptide plays an important role in improvement of the secretory capacity of overexpressed glucoamylase. However, the molecular weight of overexpressed glucoamylase is higher than that of the commercial one (around 75 kDa, the major one) and the one from *A. terreus* [27], which might be due to overglycosylation.

**Figure 4:**
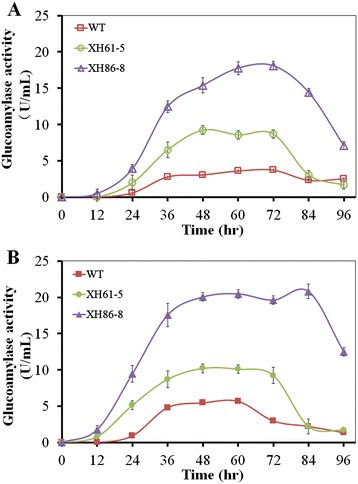
**Comparison of the effects of signal peptides on glucoamylase activity during the course of growth.** WT and the transformants XH61-5 and XH86-8 were compared in the one-step **(A)** and two-step **(B)** processes using liquefied corn starch as the carbon source. Cultures were sampled every 12 h. The activity of expressed secreted glucoamylase was assayed at 40°C and pH4.6 using soluble starch as the substrate. Specific activity was defined as one unit of enzyme activity per ml of crude culture filtrates.

As far as the glucoamylase activity is concerned (Figure 4), no matter which process was utilized, the relative activities of glucoamylase produced by three strains were as follows: XH86-8 > XH61-5 > WT, and the glucoamylase activity in the two-step process is slightly higher than in the one-step process. For XH86-8, the glucoamylase activity reached a plateau (around 20 u/ml) between 36 hr and 84 hr in the two-step process and around 15 u/ml between 48 hr and 84 hr in the one-stage process. For XH61-5, the glucoamylase activity showed a plateau between 36 hr and 72 hr in both processes. In the case of WT, the glucoamylase activity had plateaus between 36 hr and 72 hr in the one-stage process and between 36 hr and 60 hr in the two-stage one. The specific glucoamylase production rates were determined (Additional file 10: Table S1), indicating that the new signal peptide had significant effects on enhancement of the glucoamylase activity, which could possibly due to the improved secretory capacity of overexpressed glucoamylase. However, the results of SDS-PAGE and glucoamylase activity assays for WT, XH61-5 and XH86-8 were not consistent (Additional file 13: Figure S12; Figure 4). The inconsistency could possibly arise from overglycosylation and/or improper folding of overexpressed glucoamylase and/or substrate feeback regulation, which might have some negative effects on glucoamylase activity.

## Discussion

Citric acid can be directly produced from corn and potato starch by some mutant strains of *A. niger* due to high-level secretion of glucoamylase [10,11]. Moreover, a fusant strain between *A. terreus* and *A. usamii* (a glucoamylase producer) could directly produce itaconic acid from soluble starch, but the itaconate titer and production rate are too low to be used for industrial production [12]. These results suggested that glucoamylases play key roles in direct production of citrate and itaconate from starch.

In this study, direct production of itaconic acid by genetically engineered *A. terreus* from liquefied starch has been achieved through overexpression of the glucoamylase gene *glaA* in *A. terreus* under the control of the native *gpd* promoters. Our results also indicated that the parental strain could directly produce itaconic acid from liquefied corn starch, but the titer was too low compared with the *glaA* transformants. It has been reported that glucoamylase production from *A. terreus* could be induced by rice bran, maltose, and cellobiose, and was successfully purified from the culture filtrate of *A. terreus* [27,28]. However, the glucoamylase titer produced by *A. terreus* was not as high as *A. niger*, which is not sufficient for direct production of itaconate from liquefied starch by *A. terreus*.

The major protein, ATEG_02176, secreted by *A. terreus* CICC 40205 under the itaconate production conditions was identified to be an acid phosphatase precursor. Considering that itaconic acid is generally produced under the acidic and phosphate limited conditions [1], which would presumably lead to the decreased ATP level and energy charge of the cell, it might be reasonable that acid phosphatase is required to release more phosphate and is the major extracellular protein of *A. terreus* CICC 40205 under the optimal itaconate production conditions. However, Han *et al.* observed that oryzin (ATEG_03900) and ATEG_07481 (a predicted protein) were the most abundant extracellular proteins of *A. terreus* grown at pH 7.3 on different carbon sources: sucrose, glucose, and starch [29]. ATEG_02176 was not among 82 identified proteins, which might be due to the fact that it is an acid phosphatase precursor. It has been observed that pH had great effects on the extracellular proteome [30,31].

Higher itaconate production level from liquefied corn starch was obtained under the control of the signal peptide of ATEG_02176, and the results of SDS-PAGE, changes of residual glucose and oligosaccharides, and glucoamylase activity assays were consistent with this. The observed results could presumably arise from enhanced secretion of overexpressed glucoamylase by the stronger signal peptide of ATEG_02176. Some examples have demonstrated that protein secretion could be improved through using the strong signal peptides or engineering them. For instance, enhanced secretion of *Candida antarctica* lipase B with its native signal peptide in *Pichia pastoris* was achieved [32]. Ng *et al.* improved protein secretion from *Lactococcus lactis* through engineering the native signal peptide of a major secreted lactococcal protein, Usp45 [33].

The two-stage process, including the vegetative and fermentation phase, showed higher itaconate production level than the one-stage process, demonstrating that the two-stage process is beneficial for itaconate production, and changes of residual glucose and oligosaccharides, SDS-PAGE and glucoamylase activity assays also supported the conclusion. Under the optimized fermentation conditions, the best genetically engineered itaconate producer XH86-8 produced 77.6 g/L itaconic acid from liquefied corn starch after 3 days of cultivation, very close to the industrial level (~80 g/L) from saccharified starch hydrolysates [4,7], and significantly than that of the fusant between *A. terreus* and *A. usamii* (a glucoamylase producer) [12].

Thus, an efficient microbial cell factory for direct production of itaconic acid from liquefied corn starch has been established. Further improvement of the strain XH86-8 and optimization of the cultivation conditions, including medium compositions, might lead to further enhanced itaconic acid production from liquefied starch.

## Conclusions

In conclusion, *A. terreus* was successfully genetically engineered to directly produce itaconic acid from liquefied corn starch through overexpressing the glucoamylases gene. The itaconate titer was increased by replacing the glucoamylase’s native signal peptide with the stronger one. By using a two-step fermentation process, the itaconic acid titer of the best genetically engineered itaconate producer reached 77.6 g/L from liquefied corn starch, which is comparable to the production level from saccharified starch hydrolysates in industry. An efficient microbial cell factory for direct production of itaconic acid from liquefied corn starch has been established, which will be beneficial for biotechnological production of itaconic acid.

## Methods

### Materials

Chemicals and trypsin were from Sigma (St. Louis, Missouri, USA), Merck (Whitehouse Station, New Jersey, USA) or Ameresco (Framingham, Massachusetts, USA). Oligonucleotides were synthesized by Shanghai Sangon Biotech Co. Ltd (China). *Taq* and *pfu* DNA polymerases, RevertAid Reverse Transcriptase, restriction endonucleases were from Fermentas (Pittsburgh, Pennsylvania, USA) or New England BioLabs (Ipswich, Massachusetts, USA). The kits used for molecular cloning were from Omega Bio-tek Biotechnology (Norcross, Georgia, USA). Difco™ Potato Dextrose Agar was from BD. The cloning vector pMD18-T-simple was purchased from Takara Biotechnology (Otsu, Shiga, Japan). Trizol was from Invitrogen Life Technologies Corporation (Grand Island, New York, USA). Hygromycin B was obtained from Solarbio Science Technology Co., Ltd (China). ReadyStrip™ IPG Strips and Bio-Lyte 3/10 Ampholyte were from Bio-Rad (Hercules, California, USA). Corn starch was purchased from a local flour mill (Qingdao, China). α-Amylase (SUHONG AA Plus) and glucoamylase (SUHONG GA II) were obtained from Suzhou Hongda Enzyme Co., Ltd (China).

Liquefied corn starch was prepared following the recommendation of the enzyme manufacturer. Calcium chloride (0.05%, w/v) was dissolved in hot water at 60°C, then corn starch was added at 1 to 1 ratio (w/v), and pH was adjusted to 6.0. α-Amylase (0.05%, v/w, enzyme/starting material) was supplied, and cornstarch suspension was subsequently sprayed at 106-108°C for 1–2 min, then maintained at 97-98°C for about 1.5 hr (the DE (dextrose equivalent) value of liquefied corn starch is around 20). The slurry was then cooled to 60-62°C, and pH was adjusted to pH 4.0. Finally, glucoamylase (0.04%, v/w, enzyme/starting material) was added, and hydrolysis was carried out at 60-62°C for 27–28 hr.

### Plasmids, strains, media and cultivation conditions

The plasmid pSGF957 was kindly provided by Professor Kim from Seoul National University [34]. *Escherichia coli* DH5α was used for routine DNA transformation and plasmid isolation, and grown in Luria-Bertani broth at 37°C. *A. terreus* CICC 40205 from China Centre of Industrial Culture Collection was used in this study. Spores were harvested from 7-day-old potato dextrose agar plate (PDA). Cultivation in shake flasks was carried out in 500 ml non-baffled shake flask containing 55 ml itaconic acid production medium (IPM) on a rotary shaker at 200 rpm and 37°C [35].

### DNA manipulations

General molecular biology techniques were carried out following the standard procedures [36].

The 5’ upstream region of the *citA* gene (ATEG_07990) (*PcitA1*, 1878 bp from the start codon) was amplified using genomic DNA of *A. terreus* CICC 40205 as the template and the primers PcitA-F1/PcitA-R (Additional file 14: Table S2), and sequenced. The truncated promoters *PcitA2* (1121 bp), *PcitA3* (610 bp) and *PcitA4* (262 bp) were amplified from *PcitA1*using the primers PcitA-F2/PcitA-R, PcitA-F3/PcitA-R, and PcitA-F4/PcitA-R respectively. The candidate promoters were cloned into the vector pXH2-1 at the restrictions sites of *Xho*I and *Bst*BI (Additional file 15: Table S3) [16].

The glucoamylase gene *glaA* with its native signal peptide was amplified from the cDNA of *A. niger* Co827 using the primers glaA-F1 and glaA-R1, and cloned into the vectors pXH43and pXH44 (Additional file 5: Figure S5) to obtain the *glaA* expression cassettes pXH61 and pXH59 respectively (Table 1). pXH43 and pXH44 were constructed as follows: The DNA fragment of *TtrpC* was amplified by PCR from pXH2-1 using the primers TtrpC-F/TtrpC-R, then digested with *BstB*I and *Cla*I, and cloned into pXH33 and pXH34 cut with *BstB*I respectively (Additional file 15: Figure S3). Thus, the plasmids pXH43 and pXH44 containing the restriction sites of*Bgl*II, *Hind*III, and *Not*I in the MCS (Multiple Cloning Site) were constructed separately. The direction of inserted *TtrpC* was confirmed by PCR using primers PcitA-F4/TtrpC-R. The signal peptide of glucoamylase was predicted by SignalP 3.0 (http://www.cbs.dtu.dk/services/SignalP/).

### Evaluation of the*PcitA* promoters

Bright field and fluorescent images of the selected transformants at the stage of conidia, young hyphae, and mature hyphae, were taken on fluorescence microscope (Olympus BX51). Young and mature hyphae were obtained by cultivation in shake flasks at 37°C for 11 hr and 36 hr respectively.

The fluorescence intensity of two randomly selected transformants for each promoter was determined according to a published procedure [16].

### Selection of the strong signal peptide and construction of the expression cassette

The extracellular proteins of *A. terreus* CICC 40205 were prepared as previously described by Han *et al.* [29], and analyzed by two-dimensional gel electrophoresis (2-DE) according to the manufacturer’s recommendations. The main protein band was excised and treated with trypsin following the standard protocol. Protein identification and peptide mass analysis were carried out by HPLC–ESI-MS/MS. The database of Aspergillus from NCBI (http://www.ncbi.nlm.nih.gov/) was used to identify the target protein. The signal peptide of the identified protein was predicted using SignalP 3.0.

The oligonucleotides encoding the signal peptide of the identified protein were synthesized and cloned into the pMD18-T-simple vector to obtain pXH84 (Additional file 16: Figure S13). The glucoamylase gene *glaA* without its own signal peptide was amplified by PCR using the primers glaA-F2 and glaA-R and cloned into pXH84at the restriction sites of *Spe*I and *Bgl*II to obtain pXH85 (Additional file 16: Figure S13). The glucoamylase gene *glaA* containing the signal peptide of ATEG_02176 (the identified protein) named as *SP*_*02176*_*-glaA1* was amplified from pXH85 by the primers M13-47 and M13-48, and cloned into the vector pXH43 at the restriction sites of *Bst*BI and *Bcl*I (*Bgl*II) to get the expression cassette pXH86 containing *SP*_*02176*_*-glaA1* (Table 1).

### *A. terreus* transformation and analysis of itaconic acid production

The linearized expression cassettes pXH31, pXH32, pXH33, pXH34, pXH59, pXH61, and pXH86 by *Dra*I were transformed into *A. terreus* CICC 40205 respectively (Table 1). Transformants were selected on the PDA-SH plates (PDA supplemented with 1.2 M sorbitol and 100 mg/L hygromycin B). The integrations of the target genes into the genome were confirmed by genomic PCR using the primers M13-47 and GFP-seqRor glaA-R.

The transformants of pXH59, pXH61, and pXH86, which can grow healthily on the PDA plates, were first tested for itaconic acid production using saccharified corn starch hydrolysates (140 g/L glucose equivalent) as the starting material on a rotary shaker at 37°C for 72 h. The shaking diameter of the rotary shaker is 9 cm. The better itaconate producers were further screened using liquefied corn starch (corresponding to 140 g/L glucose) as the carbon source, which was prepared according to the published procedure [37]. 2.5 × 10^7^ Spores were directly inoculated in 55 ml IPM. Each transformant was grown in two or three individual flasks, and the screening experiments for the better transformants were repeated once.

The titer of itaconic acid produced was determined by HPLC (High Performance Liquid Chromatography) using an Aminex HPX-87-H column (300 mm × 7.8 mm) detected at 210 nm. The column was operated at 35°C with a mobile phase of 4 mM H_2_SO_4_ at a flow rate of 0.6 ml/min. Authentic itaconic acid was used as a standard to calculate the final concentration of itaconic acid.

The evaporation effects were taken into account when determining the itaconate and other concentrations. To maintain humidity and minimize water evaporation, water was sprayed regularly.

### Optimizing the fermentation conditions and comparison of WT and the transformants XH61-5 and XH86-8

The optimized fermentation was carried out in a two-step process, involving the vegetative (growing a pre-culture on saccharified corn starch hydrolysates) and production phase (liquefied corn starch used as the carbon source for further fermentation) [18]. 2.5 × 10^7^ Spores were first inoculated in 55 ml IPM (2% glucose equivalent as the carbon source) with 500 ml shake flasks for 16 hr at 37°C, 200 rpm, and 5 ml seed culture was then transferred into 50 ml IPM (liquefied corn starch as the carbon source, equivalent to 140 g/L glucose), and cultivated for 72 hr at 37°C, 200 rpm.

WT and the transformants XH61-5 and XH86-8 were directly compared in the one-step and two-step processes using liquefied corn starch (equivalent to 140 g/L glucose) as the carbon source. Three independents experiments were set for each strain. All assays were done at least in duplicate.

Mycelial dry weight was determined as described previously [38].

The activity of overexpressed glucoamylase was assayed following a standard procedure [39]. One unit of enzyme is defined as producing 1 mg of glucose per hour at 40°C and pH4.6. Glucoamylase activity was defined as one unit of enzyme activity per ml of enzyme or crude culture filtrates.

SDS-PAGE was directly performed for the culture filtrate of *A. terreus* following the protocol [36].

Residual glucose was quantified using the biosensor (SBA-40C) from Biology Institute of Shandong Academy of Science (Jinan, China). Residual oligosaccharides were quantified following a published procedure with slight modification (only glucoamylase was used) [40], and expressed as residual glucose equivalent, which is the amount of glucose equivalent after full hydrolysis of residual oligosaccharides minus the amount of residual glucose determined above.

## Additional files

Additional file 1: Figure S1. Bright field and fluorescent images of the transformant XH31-1. Bright field and fluorescent images of the transformant XH31-1 at the stage of conidia (A), young hyphae (B), and mature hyphae (C), were taken on fluorescence microscope (Olympus BX51). Young and mature hyphae were obtained by cultivation in shake flasks at 37°C for 11 hr and 36 hr respectively. *Scale bar* 10 μm. Additional file 2: Figure S2. Plasmid map of pXH33/pXH34. *sgfp*: the gene encoding synthetic green fluorescent protein. *TtrpC*: *A. nidulans trpC* terminator. *hph*: hygromycin B-resistant gene. Ap^r^: ampicillin resistance. Additional file 3: Figure S3. Bright field and fluorescent images of the transformants XH32-1 (A), XH33-1 (B), and XH34-1 (C). Bright field and fluorescent images of the selected transformant at the stage of conidia (a), young hyphae (b), and mature hyphae (c), were taken on fluorescence microscope (Olympus BX51). Young and mature hyphae were obtained by cultivation in shake flasks at 37°C for 11 hr and 36 hr respectively. *Scale bar* 10 μm. Additional file 4: Figure S4. Comparison of fluorescence intensity of transformants with different promoters. Two transformants for each promoter were randomly chosen. The fluorescence intensity of transformants with different promoter was determined according to the published procedure [16]. Additional file 5: Figure S5. Plasmid map of pXH43/pXH44. *TtrpC*: *A. nidulans trpC* terminator. *hph*: hygromycin B-resistant gene. Ap^r^: ampicillin resistance. Additional file 6: Figure S6. Itaconic acid production from saccharified corn starch hydrolysates by the transformants of pXH61 and pXH59. The transformants of pXH59 and pXH61were tested for itaconic acid production using saccharified corn starch hydrolysates (140 g/L glucose equivalent) as the starting material on a rotary shaker at 37°C for 72 h. The itaconate titers were determined by HPLC. Additional file 7: Figure S7. Two-dimensional gel electrophoresis for the crude culture filtrate of *A. terreus*. The extracellular proteins of *A. terreus* CICC 40205 were analyzed by two-dimensional gel electrophoresis (2-DE) according to the manufacturer’s recommendations. Additional file 8: Figure S8. Itaconic acid production from saccharified corn starch hydrolysates by the transformants of pXH86. The transformants of pXH86 were tested for itaconic acid production using saccharified corn starch hydrolysates (140 g/L glucose equivalent) as the starting material on a rotary shaker at 37°C for 72 h. The itaconate titers were determined by HPLC. Additional file 9: Figure S9. Timecourses of dry biomass for XH61-5,XH86-8 and WT from liquefied corn starch. WT and the transformants XH61-5 and XH86-8 were compared in the one-step (A) and two-step (B) processes using liquefied corn starch as the carbon source. Cultures were sampled every 12 h. Mycelial dry weight was determined. Dry biomass of WT grown from saccharified corn starch hydrolysate (SCSH) was used as the reference. Additional file 10: Table S1. Fermentation data of WT and transformants XH61-5 and XH86-8. The rates of growth, specific itaconate production, specific glucose formation, and specific glucoamylase production were determined based on the growth curve, itaconate production, residual glucose, and glucoamylase activity. LCS, liquefied corn starch; SCSH, saccharified corn starch hydrolysate; ND, Not detected; DB, dry biomass. ^1^One-step, 0–48 hr; Two-step, 0–36 hr; ^2^One-step, 0–72 hr; Two-step, 0–72 hr; ^3^One-step, WT, 0–60 hr; XH61-5, 0–48 hr; XH86-8, 0–36 hr; Two-step, WT, 0–48 hr; XH61-5, 0–36 hr; XH86-8, 0–24 hr. Additional file 11: Figure S10. Timecourses of residual glucose for XH61-5, XH86-8 and WT from liquefied corn starch. WT and the transformants XH61-5 and XH86-8 were compared in the one-step (A) and two-step (B) processes using liquefied corn starch (140 g/L glucose equivalent) as the carbon source. Cultures were sampled every 12 h. Residual glucose was quantified using the biosensor. In the *inset*, changes of glucose concentrations during the initial period were expanded. Additional file 12: Figure S11. Time courses of residual glucose equivalent fo rXH61-5, XH86-8 and WT from liquefied corn starch. WT and the transformants XH61-5 and XH86-8 were compared in the one-step (A) and two-step (B) processes usingliquefied corn starch (140 g/L glucose equivalent) as the carbon source. Cultures were sampled every 12 h. Residual oligosaccharides were quantified, and expressed as residual glucose equivalent, which is the amount of glucose equivalent after full hydrolysis of residual oligosaccharides minus the amount of residual glucose. Additional file 13: Figure S12. SDS-PAGE analysis of produced glucoamylase by XH86-8 from liquefied corn starch. WT and the transformants XH61-5 and XH86-8 were directly compared in the one-step (A) and two-step (B) processes using liquefied corn starch as the carbon source. Cultures were sampled every 12 h. SDS-PAGE was performed for the culture filtrate of *A. terreus*. A: Lane 1, protein molecular weight marker; Lane 2–9, samples of 12, 24, 36, 48, 60, 72, 84, 96 hr for XH86-8; Lane 10: sample of 48 hr for WT. B: Lane 1, protein molecular weight marker; Lane 2–8, samples of 24, 36, 48, 60, 72, 84, 96 hr for XH86-8; Lane 9: sample of 48 hr for WT. Additional file 14: Table S2. Primers used in this study. The restriction enzymes sites were underlined. Additional file 15: Table S3. Some plasmids used in this study. *Pmgpd*: *Monascus purpureus gpd1* (glyceraldehyde-3-phosphate dehydrogenase) promoter. *sgfp*: the gene encoding synthetic green fluorescent protein. *TtrpC*: *A. nidulans trpC* terminator. *hph*: hygromycin B-resistant gene. *PtrpC*: *Aspergillus nidulans trpC* promoter. Ap^r^: ampicillin resistance. *SP*_*02176*_: the gene encoding the signal peptide of acid phosphatase ATEG_02176. MCS: Multiple Cloning Site (*Bst*BI, *Xho*I, *Hind*III, *Bgl*II). Additional file 16: Figure S13. Schematic diagrams of pXH84 and pXH85. *SP*_*02176*_: the gene encoding the signal peptide of acid phosphatase ATEG_02176.*glaA*: the gene encoding glucoamylase from *A. niger*.
