# Supplementary material for: Direct production of itaconic acid from liquefied corn starch by genetically engineered Aspergillus terreus
Source: Microb Cell Fact. 2014 Aug 17;13:108. doi: 10.1186/s12934-014-0108-1 (PMC4145239; doi:10.1186/s12934-014-0108-1)

Additional file 2

Figure S2 Plasmid map of pXH33/pXH34.

sgfp: the gene encoding synthetic green fluorescent protein. *TtrpC*: *A. nidulans trpC* terminator. *hph*: hygromycin B-resistant gene. Ap^r: ampicillin resistance.

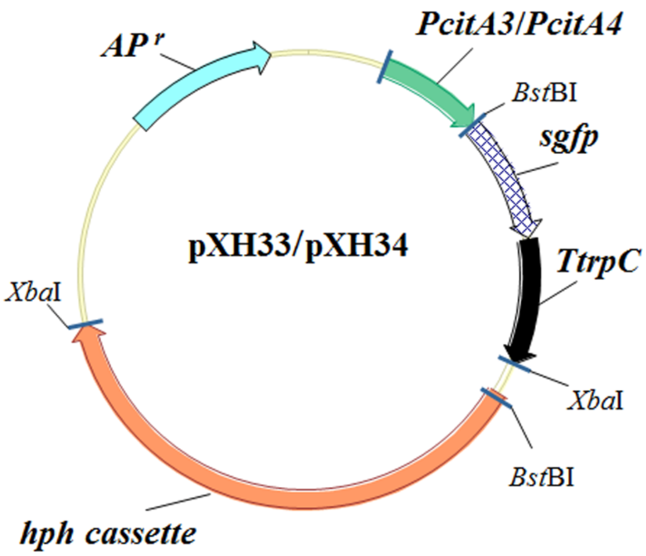

Supplement: Additional file 2: Figure S2. — Plasmid map of pXH33/pXH34. sgfp: the gene encoding synthetic green fluorescent protein. TtrpC: A. nidulans trpC terminator. hph: hygromycin B-resistant gene. Apr: ampicillin resistance. [file 12934_2014_108_MOESM2_ESM.pdf]
