# Supplementary material for: Direct production of itaconic acid from liquefied corn starch by genetically engineered Aspergillus terreus
Source: Microb Cell Fact. 2014 Aug 17;13:108. doi: 10.1186/s12934-014-0108-1 (PMC4145239; doi:10.1186/s12934-014-0108-1)

Additional file 3

Figure S3 Bright field and fluorescent images of the transformants XH32-1 (A), XH33-1 (B), and XH34-1 (C).

Bright field and fluorescent images of the selected transformant at the stage of conidia (**a**), young hyphae (**b**), and mature hyphae (**c**), were taken on fluorescence microscope (Olympus BX51). Young and mature hyphae were obtained by cultivation in shake flasks at 37 ^{o}C for 11 hr and 36 hr respectively. *Scale bar* 10 μm.

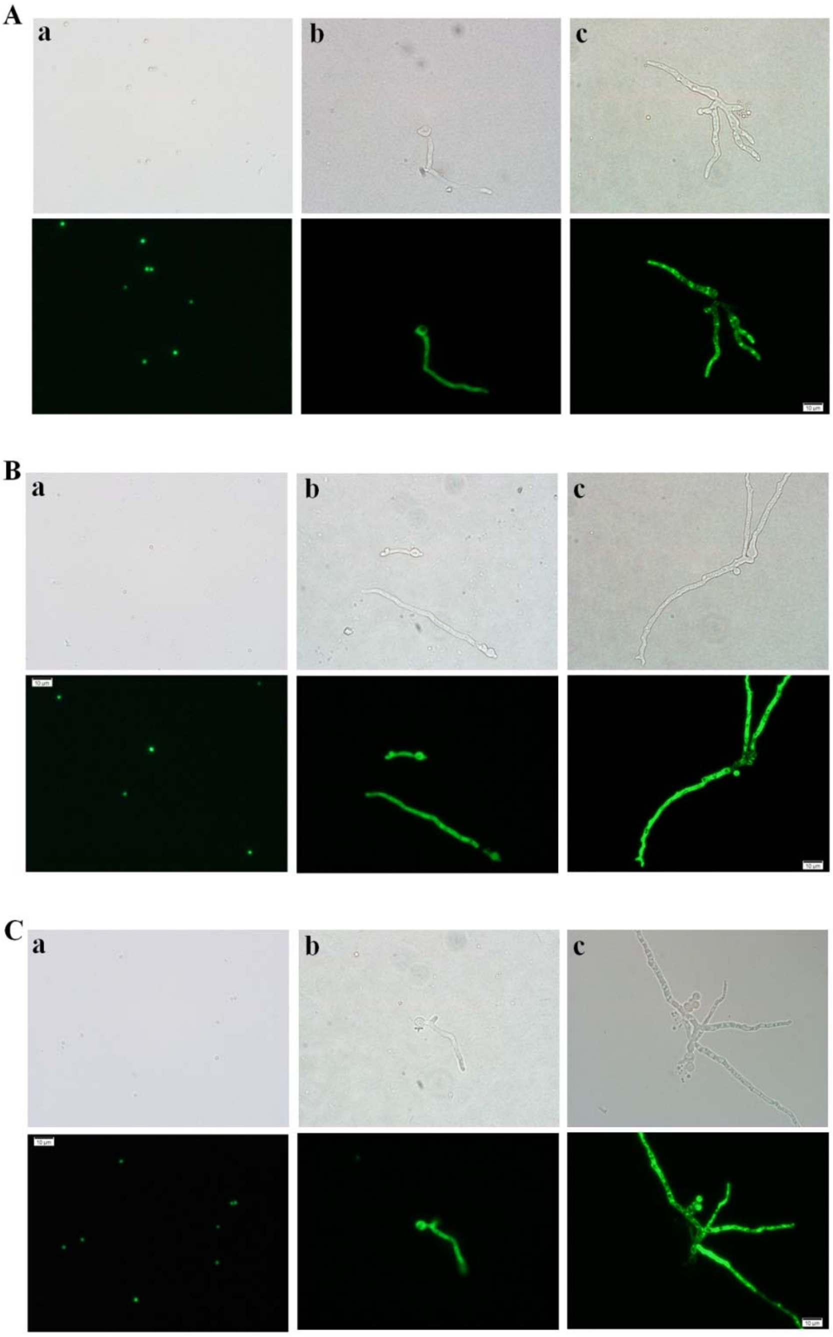

Supplement: Additional file 3: Figure S3. — Bright field and fluorescent images of the transformants XH32-1 (A), XH33-1 (B), and XH34-1 (C). Bright field and fluorescent images of the selected transformant at the stage of conidia (a), young hyphae (b), and mature hyphae (c), were taken on fluorescence microscope (Olympus BX51). Young and mature hyphae were obtained by cultivation in shake flasks at 37°C for 11 hr and 36 hr respectively. Scale bar 10 μm. [file 12934_2014_108_MOESM3_ESM.pdf]
