# Supplementary material for: Direct production of itaconic acid from liquefied corn starch by genetically engineered Aspergillus terreus
Source: Microb Cell Fact. 2014 Aug 17;13:108. doi: 10.1186/s12934-014-0108-1 (PMC4145239; doi:10.1186/s12934-014-0108-1)

Additional file 4

Figure S4 Comparison of fluorescent intensity of transformants with different promoters.

Two transformants for each promoter were randomly chosen. The fluorescence intensity of transformants with different promoter was determined according to the published procedure [16].

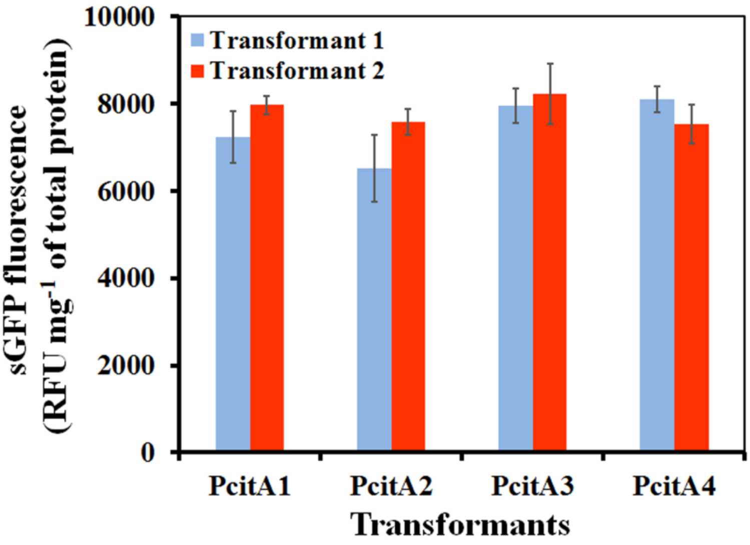

Supplement: Additional file 4: Figure S4. — Comparison of fluorescence intensity of transformants with different promoters. Two transformants for each promoter were randomly chosen. The fluorescence intensity of transformants with different promoter was determined according to the published procedure [16]. [file 12934_2014_108_MOESM4_ESM.pdf]
