# Supplementary material for: Direct production of itaconic acid from liquefied corn starch by genetically engineered Aspergillus terreus
Source: Microb Cell Fact. 2014 Aug 17;13:108. doi: 10.1186/s12934-014-0108-1 (PMC4145239; doi:10.1186/s12934-014-0108-1)

Additional file6

Figure S6 Itaconic acid production from saccharified corn starch hydrolysates by the transformants of pXH61 and pXH59.

The transformants of pXH59 and pXH61 were tested for itaconic acid production using saccharified corn starch hydrolysates (140 g/L glucose equivalent) as the starting material on a rotary shaker at 37 ^{o}C for 72 h. The itaconate titers were determined by HPLC.

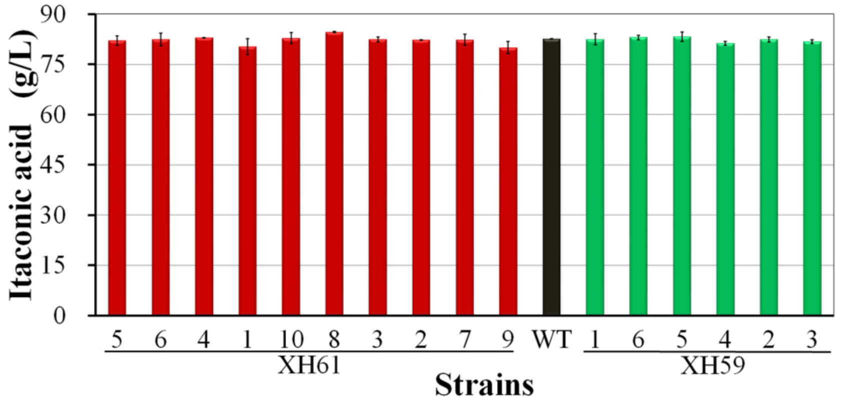

Supplement: Additional file 6: Figure S6. — Itaconic acid production from saccharified corn starch hydrolysates by the transformants of pXH61 and pXH59. The transformants of pXH59 and pXH61were tested for itaconic acid production using saccharified corn starch hydrolysates (140 g/L glucose equivalent) as the starting material on a rotary shaker at 37°C for 72 h. The itaconate titers were determined by HPLC. [file 12934_2014_108_MOESM6_ESM.pdf]
