# Supplementary material for: Direct production of itaconic acid from liquefied corn starch by genetically engineered Aspergillus terreus
Source: Microb Cell Fact. 2014 Aug 17;13:108. doi: 10.1186/s12934-014-0108-1 (PMC4145239; doi:10.1186/s12934-014-0108-1)

Additional file7

Figure S7 Two-dimensional gel electrophoresis for the crude culture filtrate of *A. terreus*.

The extracellular proteins of *A. terreus* CICC 40205 were analyzed by two-dimensional gel electrophoresis (2-DE) according to the manufacturer's recommendations.

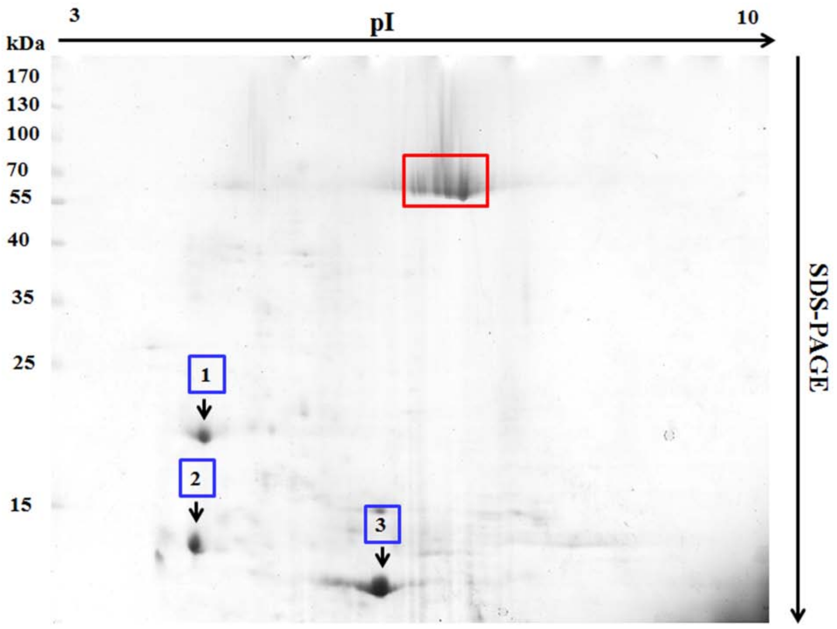

Supplement: Additional file 7: Figure S7. — Two-dimensional gel electrophoresis for the crude culture filtrate of A. terreus. The extracellular proteins of A. terreus CICC 40205 were analyzed by two-dimensional gel electrophoresis (2-DE) according to the manufacturer’s recommendations. [file 12934_2014_108_MOESM7_ESM.pdf]
