# Supplementary material for: Direct production of itaconic acid from liquefied corn starch by genetically engineered Aspergillus terreus
Source: Microb Cell Fact. 2014 Aug 17;13:108. doi: 10.1186/s12934-014-0108-1 (PMC4145239; doi:10.1186/s12934-014-0108-1)

Additional file8

Figure S8 Itaconic acid production from saccharified corn starch hydrolysates by the transformants of pXH86.

The transformants of pXH86 were tested for itaconic acid production using saccharified corn starch hydrolysates (140 g/L glucose equivalent) as the starting material on a rotary shaker at 37 ^{o}C for 72 h. The itaconate titers were determined by HPLC.

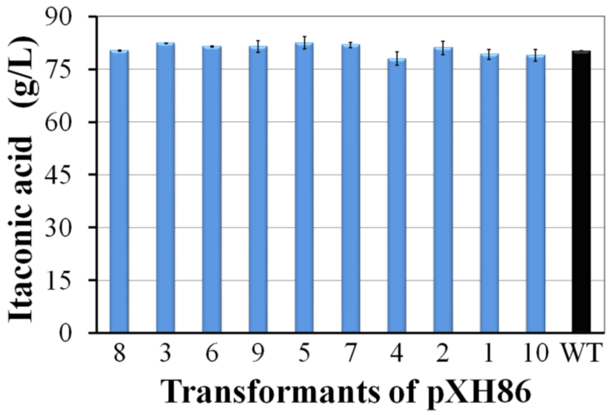

Supplement: Additional file 8: Figure S8. — Itaconic acid production from saccharified corn starch hydrolysates by the transformants of pXH86. The transformants of pXH86 were tested for itaconic acid production using saccharified corn starch hydrolysates (140 g/L glucose equivalent) as the starting material on a rotary shaker at 37°C for 72 h. The itaconate titers were determined by HPLC. [file 12934_2014_108_MOESM8_ESM.pdf]
