# Supplementary material for: Direct production of itaconic acid from liquefied corn starch by genetically engineered Aspergillus terreus
Source: Microb Cell Fact. 2014 Aug 17;13:108. doi: 10.1186/s12934-014-0108-1 (PMC4145239; doi:10.1186/s12934-014-0108-1)

Additional file 9

Figure S9 Time courses of dry biomass for XH61-5, XH86-8 and WT from liquefied corn starch.

WT and the transformants XH61-5 and XH86-8 were directly compared in the one-step (**A**) and two-step (**B**) processes using liquefied corn starch (140 g/L glucose equivalent) as the carbon source. Cultures were sampled every 12 h. Mycelial dry weight was determined. Dry biomass of WT growing from saccharified corn starch hydrolysate (SCSH) was used as the reference.

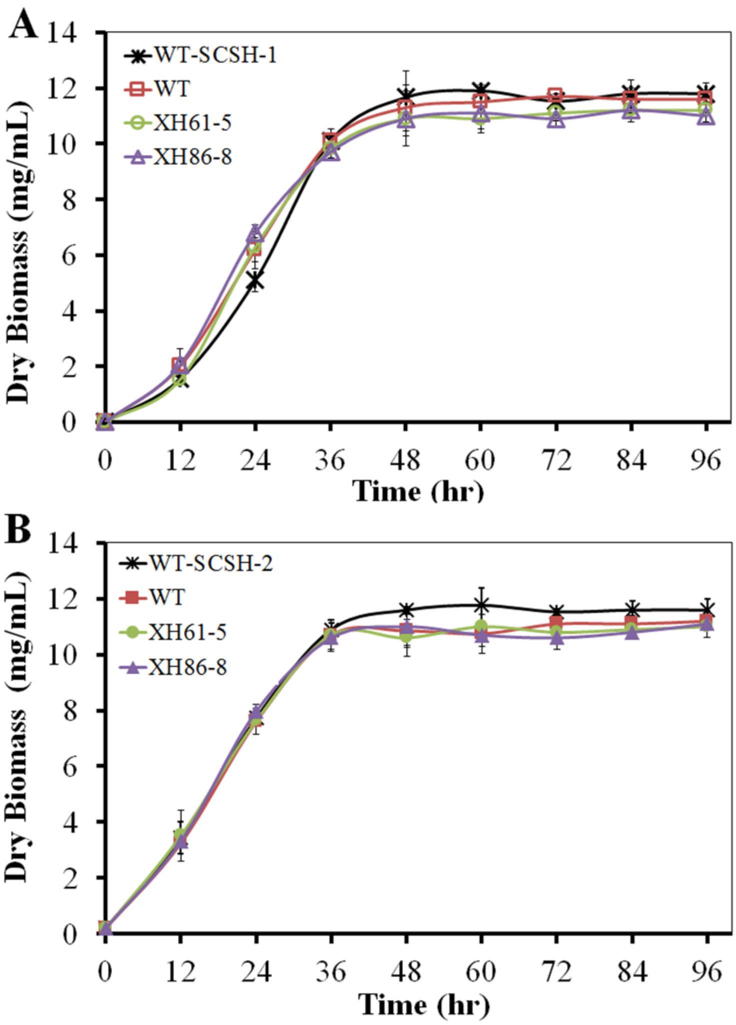

Supplement: Additional file 9: Figure S9. — Timecourses of dry biomass for XH61-5,XH86-8 and WT from liquefied corn starch. WT and the transformants XH61-5 and XH86-8 were compared in the one-step (A) and two-step (B) processes using liquefied corn starch as the carbon source. Cultures were sampled every 12 h. Mycelial dry weight was determined. Dry biomass of WT grown from saccharified corn starch hydrolysate (SCSH) was used as the reference. [file 12934_2014_108_MOESM9_ESM.pdf]
