# Supplementary material for: Direct production of itaconic acid from liquefied corn starch by genetically engineered Aspergillus terreus
Source: Microb Cell Fact. 2014 Aug 17;13:108. doi: 10.1186/s12934-014-0108-1 (PMC4145239; doi:10.1186/s12934-014-0108-1)
Supplement: Additional file 10: Table S1. — Fermentation data of WT and transformants XH61-5 and XH86-8. The rates of growth, specific itaconate production, specific glucose formation, and specific glucoamylase production were determined based on the growth curve, itaconate production, residual glucose, and glucoamylase activity. LCS, liquefied corn starch; SCSH, saccharified corn starch hydrolysate; ND, Not detected; DB, dry biomass. 1One-step, 0–48 hr; Two-step, 0–36 hr; 2One-step, 0–72 hr; Two-step, 0–72 hr; 3One-step, WT, 0–60 hr; XH61-5, 0–48 hr; XH86-8, 0–36 hr; Two-step, WT, 0–48 hr; XH61-5, 0–36 hr; XH86-8, 0–24 hr. [file 12934_2014_108_MOESM10_ESM.pdf]

Additional file 10

Table S1 Fermentation data of WT and transformants XH61-5 and XH86-8

Carbon source	Strain	Growth rate (g L^{-1} h^{-1})[1]		Specific itaconate production rate [$g(g_{DB})^{-1}$ h^{-1}][2]		Specific glucose formation rate [$g(g_{DB})^{-1}$ h^{-1}][3]		Specific glucoamylase production rate [U $(g_{DB})^{-1}$ h^{-1}][4]	
		One-step	Two-step	One-step	Two-step	One-step	Two-step	One-step	Two-step
LCS	WT	0.24±0.017	0.30±0.015	0.014±0.0019	0.072±0.0016	0.10±0.0029	0.20±0.0022	0.0056±0.0011	0.012±0.0028
	XH61-5	0.23±0.013	0.30±0.016	0.062±0.0014	0.090±0.0002	0.21±0.0043	0.30±0.0016	0.018±0.0031	0.023±0.0021
	XH86-8	0.23±0.021	0.29±0.004	0.079±0.0004	0.098±0.0002	0.37±0.0040	0.57±0.016	0.029±0.0012	0.046±0.0059
SCSH	WT	0.24±0.019	0.30±0.007	0.097±0.001	0.099±0.0007	--	--	ND	ND

LCS, liquefied corn starch; SCSH, saccharified corn starch hydrolysate; ND, Not detected; DB, dry biomass.

[1] One-step, 0-48 hr; Two-step, 0-36 hr

[2] One-step, 0-72 hr; Two-step, 0-72 hr

[3] One-step, WT, 0-60 hr; XH61-5,0-48 hr; XH86-8, 0-36 hr; Two-step, WT, 0-48 hr; XH61-5, 0-36 hr; XH86-8,0-24 hr

[4] One-step:0-48hr;Two-step:0-36 hr
