# Supplementary material for: Direct production of itaconic acid from liquefied corn starch by genetically engineered Aspergillus terreus
Source: Microb Cell Fact. 2014 Aug 17;13:108. doi: 10.1186/s12934-014-0108-1 (PMC4145239; doi:10.1186/s12934-014-0108-1)

Additional file 11

Figure S10 Time courses of residual glucose for XH61-5, XH86-8 and WT from liquefied corn starch.

WT and the transformants XH61-5 and XH86-8 were directly compared in the one-step (**A**) and two-step (**B**) processes using liquefied corn starch (140 g/L glucose equivalent) as the carbon source. Cultures were sampled every 12 h. Residual glucose was quantified using the biosensor. In the *inset*, changes of glucose concentrations during the initial period were expanded.

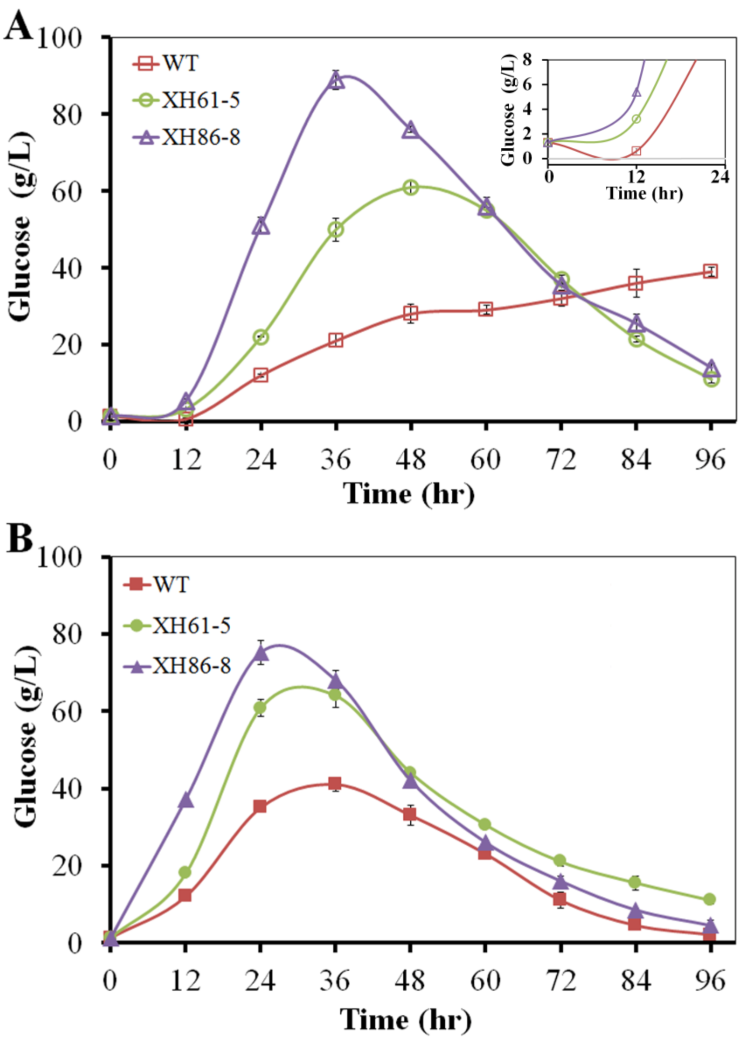

Supplement: Additional file 11: Figure S10. — Timecourses of residual glucose for XH61-5, XH86-8 and WT from liquefied corn starch. WT and the transformants XH61-5 and XH86-8 were compared in the one-step (A) and two-step (B) processes using liquefied corn starch (140 g/L glucose equivalent) as the carbon source. Cultures were sampled every 12 h. Residual glucose was quantified using the biosensor. In the inset, changes of glucose concentrations during the initial period were expanded. [file 12934_2014_108_MOESM11_ESM.pdf]
