# Supplementary material for: Direct production of itaconic acid from liquefied corn starch by genetically engineered Aspergillus terreus
Source: Microb Cell Fact. 2014 Aug 17;13:108. doi: 10.1186/s12934-014-0108-1 (PMC4145239; doi:10.1186/s12934-014-0108-1)

**Additional file 12**

**Figure S11 Time courses of residual glucose equivalent for XH61-5, XH86-8 and WT from liquefied corn starch.**

WT and the transformants XH61-5 and XH86-8 were directly compared in the one-step (**A**) and two-step (**B**) processes using liquefied corn starch (140 g/L glucose equivalent) as the carbon source. Cultures were sampled every 12 h. Residual oligosaccharides were quantified, and expressed as residual glucose equivalent, which is the amount of glucose equivalent after full hydrolysis of residual oligosaccharides minus the amount of residual glucose.

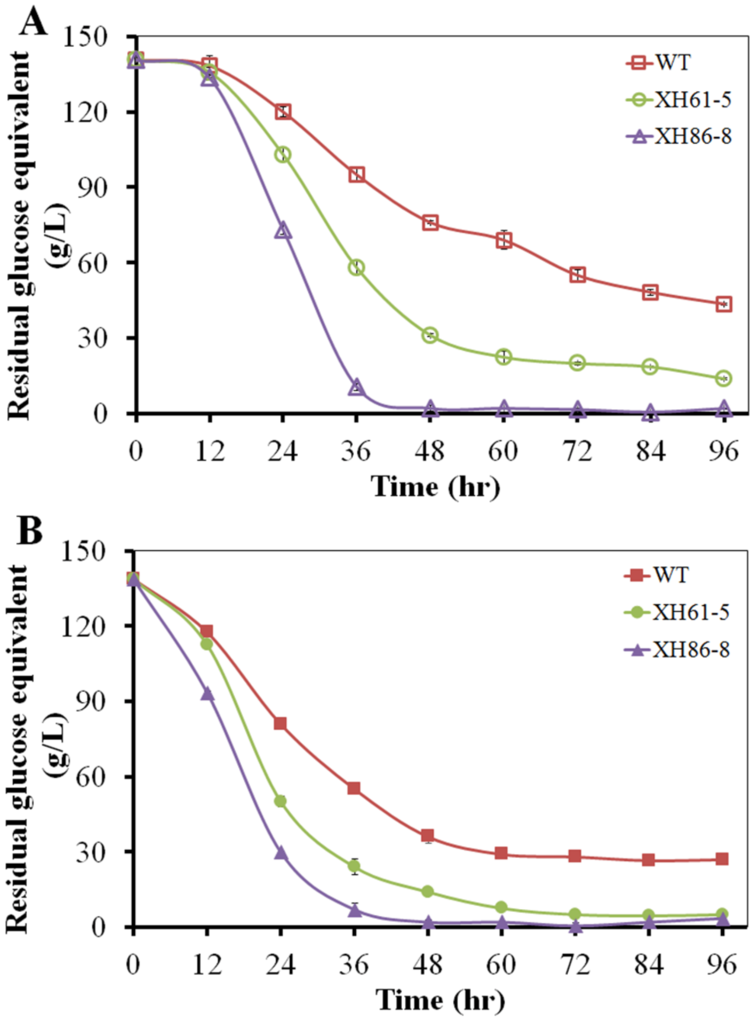

Supplement: Additional file 12: Figure S11. — Time courses of residual glucose equivalent fo rXH61-5, XH86-8 and WT from liquefied corn starch. WT and the transformants XH61-5 and XH86-8 were compared in the one-step (A) and two-step (B) processes usingliquefied corn starch (140 g/L glucose equivalent) as the carbon source. Cultures were sampled every 12 h. Residual oligosaccharides were quantified, and expressed as residual glucose equivalent, which is the amount of glucose equivalent after full hydrolysis of residual oligosaccharides minus the amount of residual glucose. [file 12934_2014_108_MOESM12_ESM.pdf]
