# Supplementary material for: Direct production of itaconic acid from liquefied corn starch by genetically engineered Aspergillus terreus
Source: Microb Cell Fact. 2014 Aug 17;13:108. doi: 10.1186/s12934-014-0108-1 (PMC4145239; doi:10.1186/s12934-014-0108-1)

Additional file 13

Figure S12 SDS-PAGE analysis of produced glucoamylase by XH86-8from liquefied corn starch.

WT and the transformants XH61-5 and XH86-8 were directly compared in the one-step (**A**) and two-step (**B**) processes using liquefied corn starch as the carbon source. Cultures were sampled every 12 h. SDS-PAGE was performed for the culture filtrate of *A. terreus*.

A: Lane 1, protein molecular weight marker; Lane 2-9, samples of 12, 24, 36, 48, 60, 72, 84, 96 hr for XH86-8; Lane 10: sample of 48 hr for WT.

B: Lane 1, protein molecular weight marker; Lane 2-8, samples of 24, 36, 48, 60, 72, 84, 96 hr for XH86-8; Lane 9: sample of 48 hr for WT.

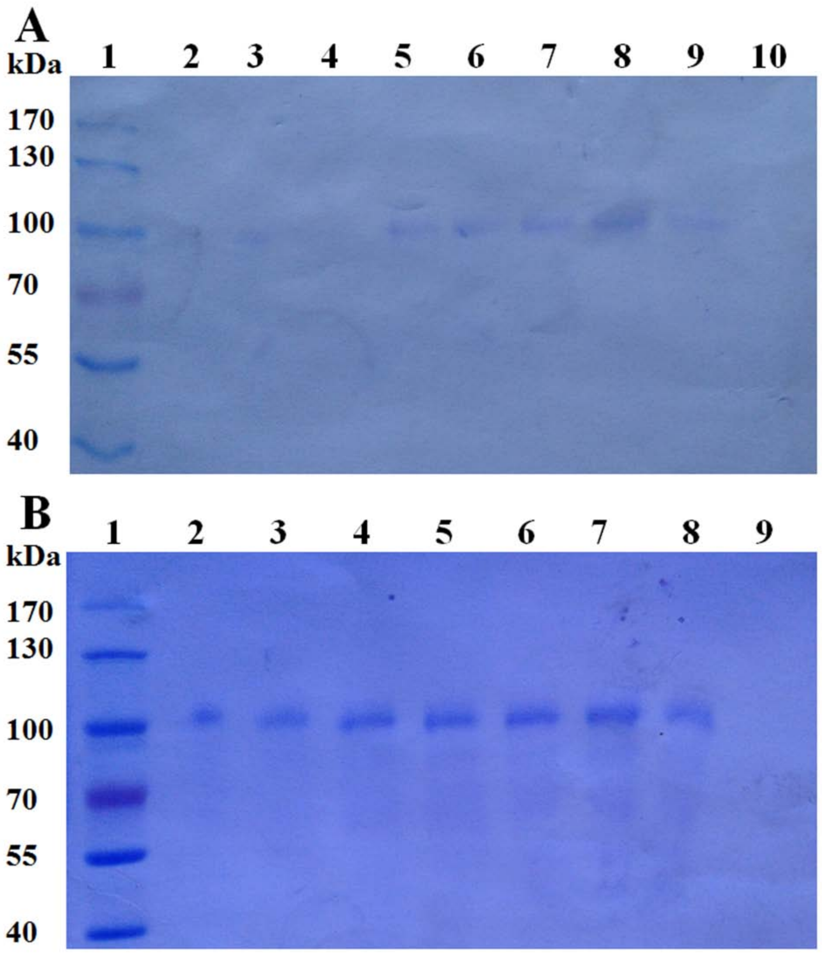

Supplement: Additional file 13: Figure S12. — SDS-PAGE analysis of produced glucoamylase by XH86-8 from liquefied corn starch. WT and the transformants XH61-5 and XH86-8 were directly compared in the one-step (A) and two-step (B) processes using liquefied corn starch as the carbon source. Cultures were sampled every 12 h. SDS-PAGE was performed for the culture filtrate of A. terreus. A: Lane 1, protein molecular weight marker; Lane 2–9, samples of 12, 24, 36, 48, 60, 72, 84, 96 hr for XH86-8; Lane 10: sample of 48 hr for WT. B: Lane 1, protein molecular weight marker; Lane 2–8, samples of 24, 36, 48, 60, 72, 84, 96 hr for XH86-8; Lane 9: sample of 48 hr for WT. [file 12934_2014_108_MOESM13_ESM.pdf]
