# Supplementary material for: Direct production of itaconic acid from liquefied corn starch by genetically engineered Aspergillus terreus
Source: Microb Cell Fact. 2014 Aug 17;13:108. doi: 10.1186/s12934-014-0108-1 (PMC4145239; doi:10.1186/s12934-014-0108-1)
Supplement: Additional file 14: Table S2. — Primers used in this study. The restriction enzymes sites were underlined. [file 12934_2014_108_MOESM14_ESM.pdf]

Additional file 14

Table S2 Primers used in this study.

Primer	Sequence (5’-3’)
PcitA-F1	CAAGCTCGAGTGGTTGGTAGTGTGGATTATG (*Xho*I)
PcitA-F2	CAAGCTCGAGCGAATCTGGCAACGTCCTCCG (*Xho*I)
PcitA-F3	GATC GTCGAC GGTACC AACCAAGGACCGCGATGACC (*Sal*I)
PcitA-F4	CATG GTCGAC GGTACC ATACTGTACCGATTTCCGCC (*Sal*I)
PcitA-R	CACGCCATGGTTCGAATCCAACAAGAGTC (*Nco*I, *Bst*BI)
GFP-seqR	CTCGGCGCGGGTCTTGTAGTTG
TtrpC-F	CAGTTCGAAATGCTCGAGCGAAGCTTAGATCTACTTAACGTT ACTGAAATC (*Bst*BI, *Xho*I, *Hin*dIII,*Bgl*II)
TtrpC-R	GTAATCGATACGAAGATCACTGGGAACAACTGGC (*Cla*I)
glaA-F1:	GACT TTCGAA ATGTCGTTCCGATCTCTACTCGC (*Bst*BI)
glaA-F2:	GACT ACTAGTAATGTGATTTCCAAGCGCGC (*Spe*I)
glaA-R	CATA AGATCT CCGCCAGGTGTCAGTCAC (*Bgl*II)
PgpdAt1-F	GACCTCGAGATATGGCCTACGAACGAATTG (*Xho*I)
PgpdAt-R1	AGCCTCTAGAAGCACATGTTGTGATGATTGATGAGTTGTTGG (*Xba*I, *Pci*I)
mtpMD18-F	GGATAACGCAGGAAAGAAGATGTGAGCAAAAGGCCAG
mtpMD18-R	CTGGCCTTTTGCTCACATCTTCTTTCCTGCGTTATCC
sgfp-F	AGCCACATGTTGAGCAAGGGCGAGGAGC (*Pci*I)
TtrpC-R	GTCGTGCCAGTCGACTCTAGA (*Xba*I)

The restriction enzymes sites were underlined.
