# Supplementary material for: Direct production of itaconic acid from liquefied corn starch by genetically engineered Aspergillus terreus
Source: Microb Cell Fact. 2014 Aug 17;13:108. doi: 10.1186/s12934-014-0108-1 (PMC4145239; doi:10.1186/s12934-014-0108-1)
Supplement: Additional file 15: Table S3. — Some plasmids used in this study. Pmgpd: Monascus purpureus gpd1 (glyceraldehyde-3-phosphate dehydrogenase) promoter. sgfp: the gene encoding synthetic green fluorescent protein. TtrpC: A. nidulans trpC terminator. hph: hygromycin B-resistant gene. PtrpC: Aspergillus nidulans trpC promoter. Apr: ampicillin resistance. SP 02176: the gene encoding the signal peptide of acid phosphatase ATEG_02176. MCS: Multiple Cloning Site (BstBI, XhoI, HindIII, BglII). [file 12934_2014_108_MOESM15_ESM.pdf]

Additional file 15

Table S3 Some plasmids used in this study

Plasmids	Relevant characteristics
pSGF957	*Pmgpd-sgfp-TtrpC*, *hph* marker (*PtrpC-hph-TtrpC*) [34]
pMD18s	The cloning vector pMD18-T-simple, Ap^r
pXH2-1	*PgpdAt1-sgfp-TtrpC*, *hph* marker
pXH43	*PcitA3-MCS-TtrpC*, *hph* marker
pXH44	*PcitA4-MCS-TtrpC*, *hph* marker
pXH84	pMD18s derivative, containing SP_{02176}, Ap^r
pXH85	pXH84 derivative, containing SP_{02176}-*glaA1* gene, Ap^r
