# Supplementary material for: Direct production of itaconic acid from liquefied corn starch by genetically engineered Aspergillus terreus
Source: Microb Cell Fact. 2014 Aug 17;13:108. doi: 10.1186/s12934-014-0108-1 (PMC4145239; doi:10.1186/s12934-014-0108-1)

Additional file 16

Figure S13 Schematic diagrams of pXH84 and pXH85.

SP_{02176}: the gene encoding the signal peptide of acid phosphatase ATEG_02176. *glaA*: the gene encoding glucoamylase from *A. niger*.

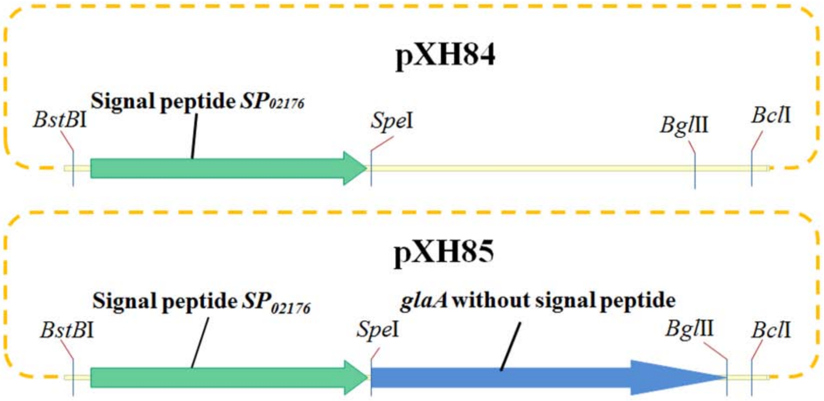

Supplement: Additional file 16: Figure S13. — Schematic diagrams of pXH84 and pXH85. SP 02176: the gene encoding the signal peptide of acid phosphatase ATEG_02176.glaA: the gene encoding glucoamylase from A. niger. [file 12934_2014_108_MOESM16_ESM.pdf]
